# A single phosphoacceptor residue in BGLF3 is essential for transcription of Epstein-Barr virus late genes

**DOI:** 10.1371/journal.ppat.1007980

**Published:** 2019-08-28

**Authors:** Jinlin Li, Ann Walsh, TuKiet T. Lam, Henri-Jacques Delecluse, Ayman El-Guindy

**Affiliations:** 1 Department of Pediatrics Yale University School of Medicine, New Haven, Connecticut, United States of America; 2 Department of Molecular Biophysics and Biochemistry, Yale University, New Haven, Connecticut, United States of America; 3 Keck MS and Proteomics Resource, Yale University, New Haven, Connecticut, United States of America; 4 Department of Tumor Virology, German Cancer Research Center, Im Neuenheimer Feld, Heidelberg, Germany; 5 Yale Cancer Center, New Haven, Connecticut, United States of America; Tulane University School of Medicine, UNITED STATES

## Abstract

Almost one third of herpesvirus proteins are expressed with late kinetics. Many of these late proteins serve crucial structural functions such as formation of virus particles, attachment to host cells and internalization. Recently, we and others identified a group of Epstein-Barr virus early proteins that form a pre-initiation complex (vPIC) dedicated to transcription of late genes. Currently, there is a fundamental gap in understanding the role of post-translational modifications in regulating assembly and function of the complex. Here, we used mass spectrometry to map potential phosphorylation sites in BGLF3, a core component of the vPIC module that connects the BcRF1 viral TATA box binding protein to other components of the complex. We identified threonine 42 (T42) in BGLF3 as a phosphoacceptor residue. T42 is conserved in BGLF3 orthologs encoded by other gamma herpesviruses. Abolishing phosphorylation at T42 markedly reduced expression of vPIC-dependent late genes and disrupted production of new virus particles, but had no effect on early gene expression, viral DNA replication, or expression of vPIC-independent late genes. We complemented failure of BGLF3(T42A) to activate late gene expression by ectopic expression of other components of vPIC. Only BFRF2 and BVLF1 were sufficient to suppress the defect in late gene expression associated with BGLF3(T42A). These results were corroborated by the ability of wild type BGLF3 but not BGLF3(T42A) to form a trimeric complex with BFRF2 and BVLF1. Our findings suggest that phosphorylation of BGLF3 at threonine 42 serves as a new checkpoint for subsequent formation of BFRF2:BGLF3:BVLF1; a trimeric subcomplex essential for transcription of late genes. Our findings provide evidence that post-translational modifications regulate the function of the vPIC nanomachine that initiates synthesis of late transcripts in herpesviruses.

## Author summary

EBV is an oncogenic virus involved in the development of about 1.5% of human cancers worldwide. EBV infection has latent and lytic forms. Both forms of infection contribute to the oncogenic capacity of the virus. During the lytic cycle, a cascade of temporally regulated events takes place leading to release of new virus particles. A crucial event in the lytic cascade is expression of the class of EBV late genes, which occurs after viral genome amplification. Late genes mainly encode virus structural proteins that are essential for virus transmission. For many years, the mechanisms regulating expression of late genes remained unknown. Recently, a set of proteins that control expression of late genes was discovered. These proteins form a unique viral pre-initiation complex (vPIC), which initiates synthesis of late gene mRNAs. To this day we have yet to fully understand the process by which assembly of vPIC is synchronized to result in a functional transcription machinery. In this report, we demonstrated that BGLF3, a component of vPIC, is modified by phosphorylation during the lytic phase of the viral life cycle. Phosphorylation of BGLF3 is essential for the ability of the protein to interact with two other components of vPIC, BFRF2 and BVLF1. Our results show that formation of the BGLF3, BFRF2 and BVLF1 complex is integral for synthesis of viral structural proteins. This report establishes the importance of post-translational modifications in regulating the function of vPIC in synthesis of herpesvirus structural proteins. Our findings have the potential to promote the discovery of new anti-viral drugs that inhibit assembly and release of oncogenic herpesviruses.

## Introduction

Lytic infection is intrinsic to the pathogenesis of herpesviruses. Virus particles are synthesized and assembled exclusively during the lytic phase. The lytic phase of oncogenic gamma herpesviruses contributes to tumor development by expanding the population of latently infected cells that possess the potential to become neoplastic. Lytic gene products also encode and upregulate expression of inflammatory cytokines, anti-apoptotic proteins, signaling molecules, and immunoevasins that promote cell proliferation and suppress immune recognition.

Temporal control of expression of lytic viral genes, a common theme among all herpesviruses, can be categorized into pre- and post-replication events. Mechanisms that regulate expression of these two classes of viral genes are quite distinct. Pre-replication genes, referred to as early genes, are regulated in a manner similar to that of cellular genes. Early gene promoters encompass multiple binding sites for transcription factors that facilitate recruitment of the basic transcription machinery. Post-replication genes, referred to as late genes, have unique promoter structures featuring a non-canonical TATA box element (reviewed in [1, 2]). Activation of late promoters is dependent on amplification of the viral genome. The strict dependence of late gene expression on replication of the viral genome represents one of the longstanding conundrums in the biology of DNA viruses.

Major progress in our current understanding of regulation of late gene expression resulted from identifying a group of lytic herpesvirus proteins that function as late gene transcription regulators [3–5]. This group of late gene regulators is conserved among beta and gamma herpesviruses [6–12]. We and others identified seven EBV proteins as essential for expression of late genes. These EBV late gene regulators are: BcRF1 (viral TATT box binding protein, vTBP), BDLF3.5, BDLF4, BFRF2, BGLF3, BGLF4 (viral protein kinase) and BVLF1 [13–18]. The current model suggests that late gene regulators assemble to form a viral pre-initiation complex (vPIC) on late promoters [1, 13]. Using specific siRNAs to all seven late gene regulators combined with RNA-seq of EBV gene transcripts, we demonstrated that a subgroup of late viral genes is transcribed in a manner independent of vPIC [19]. This phenomenon was confirmed by other groups using different approaches including CAGE-seq analyses [20, 21]. Two of these vPIC-independent late genes encode viral immunoevasins, BCRF1 (viral IL10) and BPLF1 (viral deubiquitinase) [19]. This new insight demonstrates the presence of distinct mechanisms for expression of EBV late structural proteins (vPIC-dependent) versus expression of late viral immunoevasins (vPIC-independent). The mechanism by which vPIC-independent late genes (viral IL10 and viral deubiquitinase) are transcribed is yet to be characterized.

While expression of all components of vPIC occurs during the early phase of the lytic cycle, transcription of late genes is nonetheless dependent on viral DNA replication. Recent reports demonstrated that late transcripts are synthesized from newly replicated viral genomes and require continuous genome amplification [17, 22]. Relatively little is known about the exact function of the various components of vPIC in transcription of late genes. Several late gene regulators have no identifiable domains or cellular homologs (e.g. BDLF4, BDLF3.5, BGLF3, BFRF2, and BVLF1). BcRF1, a viral protein predicted to have a saddle-like structure that is characteristic of the cellular TATA-box binding protein (TBP) [10], selectively recognizes late promoters by binding to a non-canonical TATA box element (TATT) [14, 23]. To understand the role of individual proteins in transcription of late genes, several protein interactions were identified between components of vPIC and subunits of RNAPII. BcRF1 and its orthologs of vTBPs in beta and gamma herpesviruses interact with several subunits of RNA polymerase II (RNAP II); a step considered necessary to recruit RNAPII complex to late promoters [4, 13, 24]. Davis et al mapped the motif interacting with RPB1, RNAPII catalytic subunit, to three leucine residues at the N-terminal domain of ORF24 (the ortholog of EBV vTBP BcRF1) [24]. HCMV UL79, the ortholog of EBV BVLF1, also interacts with multiple subunits of RNAPII. These interactions augment the transcriptional activity of RNAPII suggesting a role in transcript elongation [25]. A number of additional protein-protein interactions were mapped among the various components of vPIC that provide insight into the general organization of the whole complex. For example, BVLF1 orthologs in KSHV (ORF18) and CMV (UL79) form crucial interactions with their corresponding BDLF3.5 orthologs [6, 11]. Whether these interactions are necessary for the role of BVLF1 orthologs in promoting the elongation activity of RNAPII is yet to be determined. In addition, the KSHV ortholog of BGLF3 (ORF34) serves as a core component; the protein physically interacts with four other members of vPIC and is thought to serve as a bridge between vTBPs and the rest of the complex [12, 26]. Mutations that disrupt interaction of KSHV TBP (ORF24) with the KSHV ortholog of BGLF3 (ORF34) abolished synthesis of late transcripts [26].

Despite the significant progress made towards comprehending the organization of vPIC, the role of post-translational modifications leading to assembly, regulation, and function of vPIC need to be addressed to gain better understanding of the dynamics of the complex. Here, we asked whether phosphorylation regulates the function of vPIC in transcription of late genes. Phosphorylation regulates many primary biological processes in eukaryotic cells, such as cell division, DNA replication, transcription, differentiation, and apoptosis. To address the role of phosphorylation in regulating late gene expression, we studied the phosphorylation state of BGLF3. We found that BGLF3 is phosphorylated *in vivo* at threonine 42. Phosphorylation of BGLF3 is essential for transcription of vPIC-dependent late genes. Our findings indicate that phosphorylation of BGLF3 regulates the capacity of the protein to form a trimeric complex with two other late gene regulators, BFRF2 and BVLF1. Formation of this trimeric complex is crucial for expression of late genes.

## Results

### BGLF3 is phosphorylated *in vivo* at threonine 42

**Fig 1:**
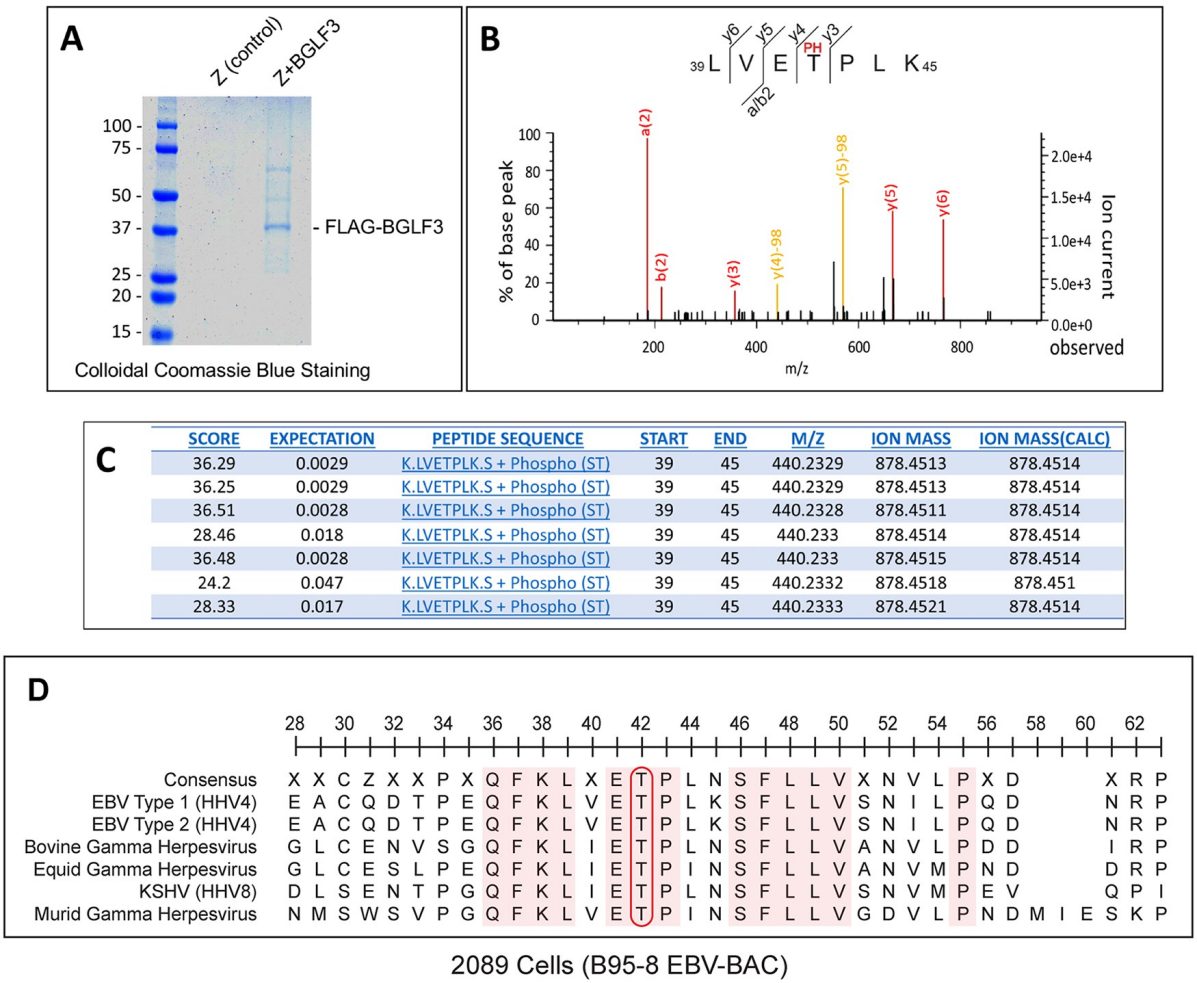
Identification of BGLF3 threonine 42 as a phosphoacceptor site. EBV positive 2089 cells were transfected with ZEBRA or ZEBRA plus FLAG-tagged BGLF3. Cells were harvested after 48h and cell lysates were subjected to immunoprecipitation (IP) using agarose beads coated with anti-FLAG antibody. Immunoprecipitated BGLF3 and associated proteins were eluted using 3X FLAG peptide. (A) Twenty percent of the eluted proteins was resolved on 10% SDS-PAGE and stained with colloidal Coomassie Blue. The remaining 80% was digested with trypsin and subjected to phosphoenrichment followed by LC-MS/MS. (B) A representative MS/MS spectrum of the BGLF3 peptide spanning amino acids 39 to 45 and its observed fragmentation pattern. (C) A table summarizing detection of the 39 to 45 phosphopeptide present in BGLF3 in seven independent experiments. (D) Sequence alignment of the region encompassing T42 in BGLF3 and related orthologs in other gamma herpesviruses.

BGLF3 and its herpesvirus orthologs are indispensable for transcription of late viral genes encoding structural proteins [18, 19]. The exact role of BGLF3 in the process of late gene expression remains largely unknown. The KSHV ortholog of BGLF3 was shown to interact with individual components of vPIC [12]. These interactions led to the hypothesis that BGLF3 functions as a core protein that connects various components of vPIC. How BGLF3 accommodates the formation of vPIC and whether post-translational modifications regulate the capacity of the protein to mediate one or more of these interactions are questions that were not addressed previously. In this report, we first assessed whether BGLF3 is phosphorylated *in vivo* during the late phase of the EBV lytic cycle. We immunoprecipitated FLAG-tagged BGLF3 from 2089 cells co-expressing the lytic cycle activator, ZEBRA. A fraction of the immunoprecipitated BGLF3 protein was resolved on SDS-PAGE and stained with colloidal Coomassie blue stain. A distinct protein band with a molecular weight equivalent to that of BGLF3 was detected (Fig 1A). The remainder of the BGLF3 eluate was subjected to trypsin digestion followed by phosphopeptide-enrichment using TiO_2_ resins. Bound phospho-peptides were eluted and analyzed by liquid chromatography-tandem mass spectrometry (LC-MS/MS). Fig 1B shows a representative MS/MS spectrum of the single BGLF3 phospho-peptide (amino acids 39 to 45) that was reproducibly shown to be phosphorylated. Phosphorylation of this peptide was evident by the detection of fragment *y5* with and without a phosphate moiety (-98 Da). Moreover, other peptide fragments in which the phosphate group was either present or lost were also detected, *y6* and *y4*, respectively. Phosphorylation of BGLF3 was reproduced seven independent times; the 39–45 peptide was consistently identified with Mascot score greater than identity score; the expectation values of the detected 39–45 BGLF3 peptide were as follows: 0.047, 0.018, 0.017, 0.0028, 0.0028, 0.0029, and 0.0029 (Fig 1C). Sequence alignment of the motif encompassing threonine at position 42 revealed that this region is conserved in other gamma herpesviruses (Fig 1D). Based on the fragmentation pattern and the fact that threonine 42 is the only amino acid susceptible to phosphorylation in this BGLF3 peptide, we conclude that BGLF3 is phosphorylated at threonine 42 during EBV lytic infection.

### Mutation of BGLF3 at threonine 42 selectively abolishes synthesis of EBV late structural proteins

**Fig 2:**
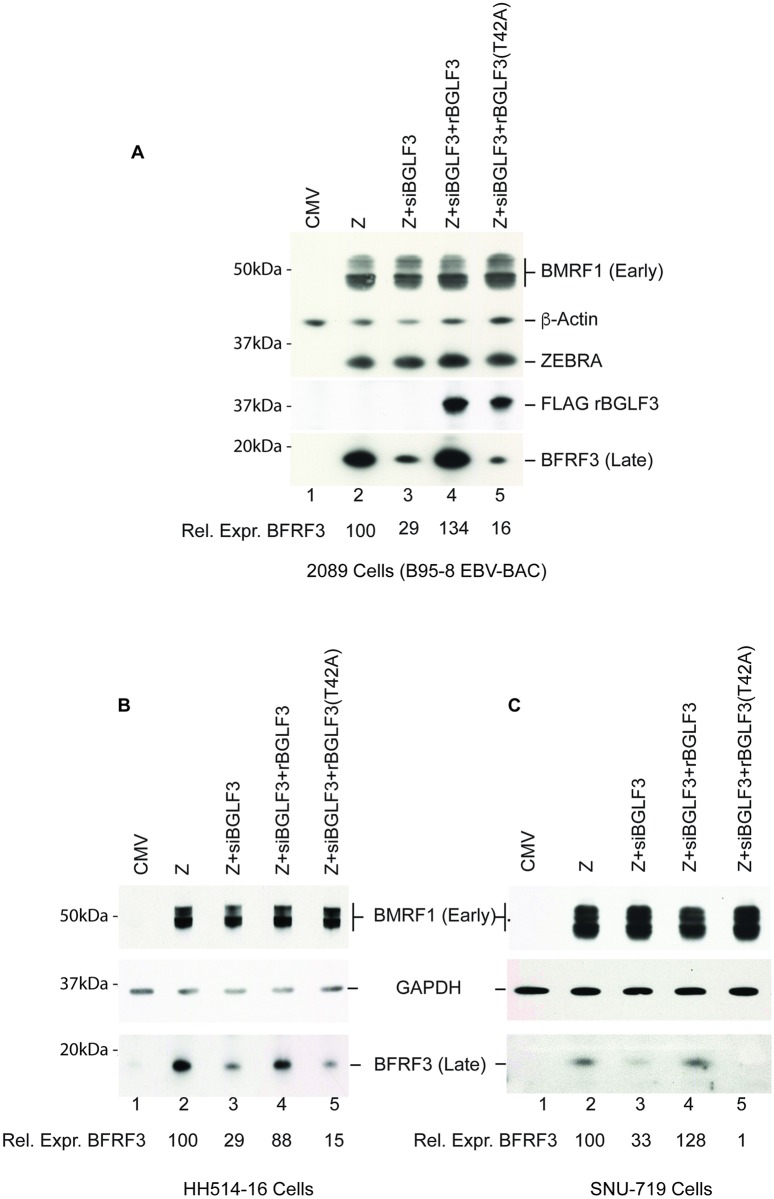
Phosphoacceptor threonine 42 is essential for expression of EBV BFRF3 capsid protein. Western blot analysis to study the effect of substituting threonine 42 in BGLF3 to alanine on viral gene expression. 2089 (A), Burkitt lymphoma HH514-16 (B) and gastric carcinoma SNU-719 (C) cells were transfected with the indicated expression vectors. Expression of endogenous BGLF3 was eliminated using siRNA to BGLF3 (siBGLF3). siRNA-resistant wild type BGLF3 (rBGLF3) and mutant BGLF3 [rBGLF3(T42A)] were provided ectopically to complement the lack of endogenous BGLF3. After 48h, cell lysates were prepared and viral protein expression was analyzed by immunoblotting using antibodies specific to the FLAG epitope, ZEBRA, β-actin, early BMRF1 and late BFRF3. The figure is a representative of three independent experiments.

We examined the functional importance of phosphorylation of BGLF3 at threonine 42 on different stages of the EBV lytic cycle, particularly late gene expression. The experiment was performed in 2089 cells induced into the lytic phase by ectopic expression of ZEBRA. In accordance with our previous data [18], knockdown of endogenous BGLF3 using specific siRNA (siBGLF3) markedly reduced expression of BFRF3 late protein, a component of the viral capsid protein (Fig 2A). To demonstrate that the effect of siBGLF3 on expression of BFRF3 was specific to silencing BGLF3 rather than an off-target activity, we inserted silent mutations to generate a form of BGLF3 that is resistant to the siRNA, referred to as rBGLF3. Ectopic expression of rBGLF3 suppressed the effect of siBGLF3 on synthesis of late products and restored expression of the late BFRF3 protein. Expressing a mutant form of rBGFL3 in which threonine 42 was mutated to alanine, rBGLF3(T42A), failed to suppress the effect of siBGLF3 on synthesis of the late BFRF3 protein. Neither knockdown of BGLF3 nor mutation of T42 had any significant effect on expression of the BMRF1 early protein, a component of the viral DNA polymerase complex (Fig 2A).

To determine whether phosphorylation of BGLF3 at threonine 42 is important for expression of late genes in physiologically relevant cell lines, we assessed the effect of the T42A mutation in HH415-16 and SNU-719 cells, which are derived from naturally EBV infected Burkitt lymphoma and gastric carcinoma, respectively. HH415-16 cells and SNU-719 cells were induced into the lytic cycle by expression of ZEBRA. Expression of endogenous BGLF3 was silenced using siBGLF3. We found that synthesis of the late BFRF3 protein was markedly reduced when threonine 42 was substituted with alanine in rBGLF3 (Fig 2B and 2C, compare lanes 4 and 5). Our results demonstrate that abolishing phosphoacceptor threonine 42 in BGLF3 is deleterious for expression of the late BFRF3 viral capsid protein in three different cell lines.

### A phosphorylatable residue at position 42 is essential for the capacity of BGLF3 to activate expression of late genes

**Fig 3:**
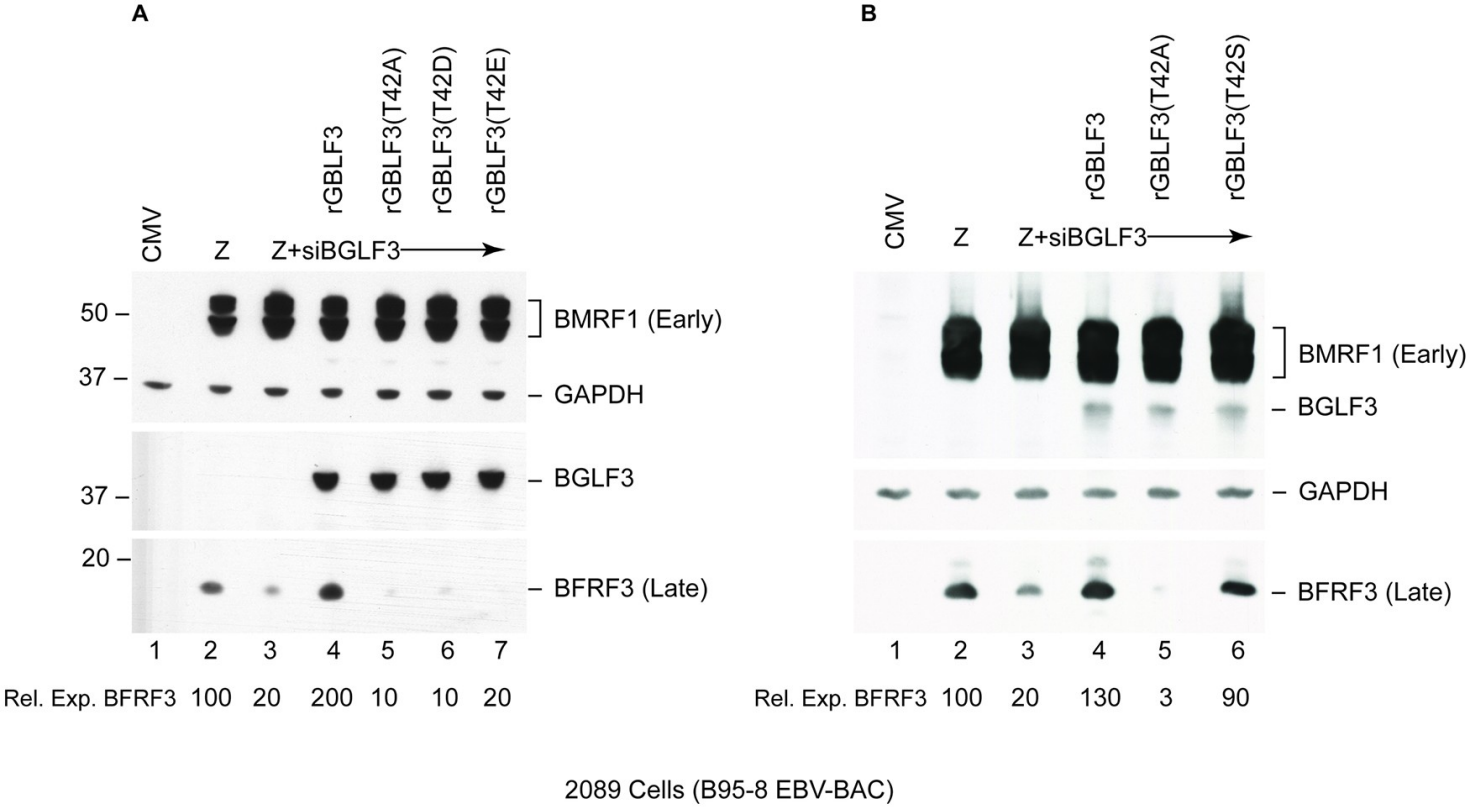
Phosphorylatable BGLF3(T42S) maintains expression of late genes. Western blot analysis assessing the effect of mutating BGLF3 at position 42 to phosphomimetic aspartate or glutamate (A) and to phosphorylatable serine (B). 2089 cells were transfected with the indicated expression vectors. Expression of endogenous BGLF3 was suppressed using siRNA to BGLF3 (siBGLF3). siRNA-resistant wild type BGLF3 (rBGLF3) was transfected to restore late gene expression. To examine the capacity of the phosphomimetic and phosphorylatale residues to restore late gene expression we transfected rBGLF3(T42D), rBGLF3(T42E) and rBGLF3(T42S). Cell lysates were analyzed after 48h by immunoblotting using antibodies specific to early BMRF1, FLAG epitope for BGLF3, GAPDH, and late BFRF3.

One approach that is commonly used to study constitutive protein phosphorylation is to mutate the phosphorylated site to a phosphomimetic residue, aspartate or glutamate. While phosphmimetic substitutions contributed to the understanding of the role of phosphorylation in many proteins, it often fails to mimic phosphorylation events that are regulated and not constitutive. To determine whether a phosphomimetic mutation would substitute for the presence of phospho-T42, we mutated T42 in rBGLF3 to aspartate (T42D) and glutamate (T42E) residues. As shown previously, ectopic expression of wild type rBGLF3 suppressed the effect of siBGLF3 and restored expression of the late BFRF3 protein. However, neither the aspartate nor glutamine substitutions lead to restoration of BFRF3 expression (Fig 3A).

To determine whether the presence of a phosphorylatable residues (e.g. serine) is sufficient to maintain expression of late genes, we mutated T42 to serine. We found that ectopic expression of BGLF3(T42S) suppressed the effect of siBGLF3 and restored expression of BFRF3 (Fig 3B). These results demonstrate that a phosphorylatable residue at position 42 is essential for the capacity of BGLF3 to mediate expression of late genes.

### Alanine substitution of BGLF3 at threonine 42 disrupts transcription of late genes

**Fig 4:**
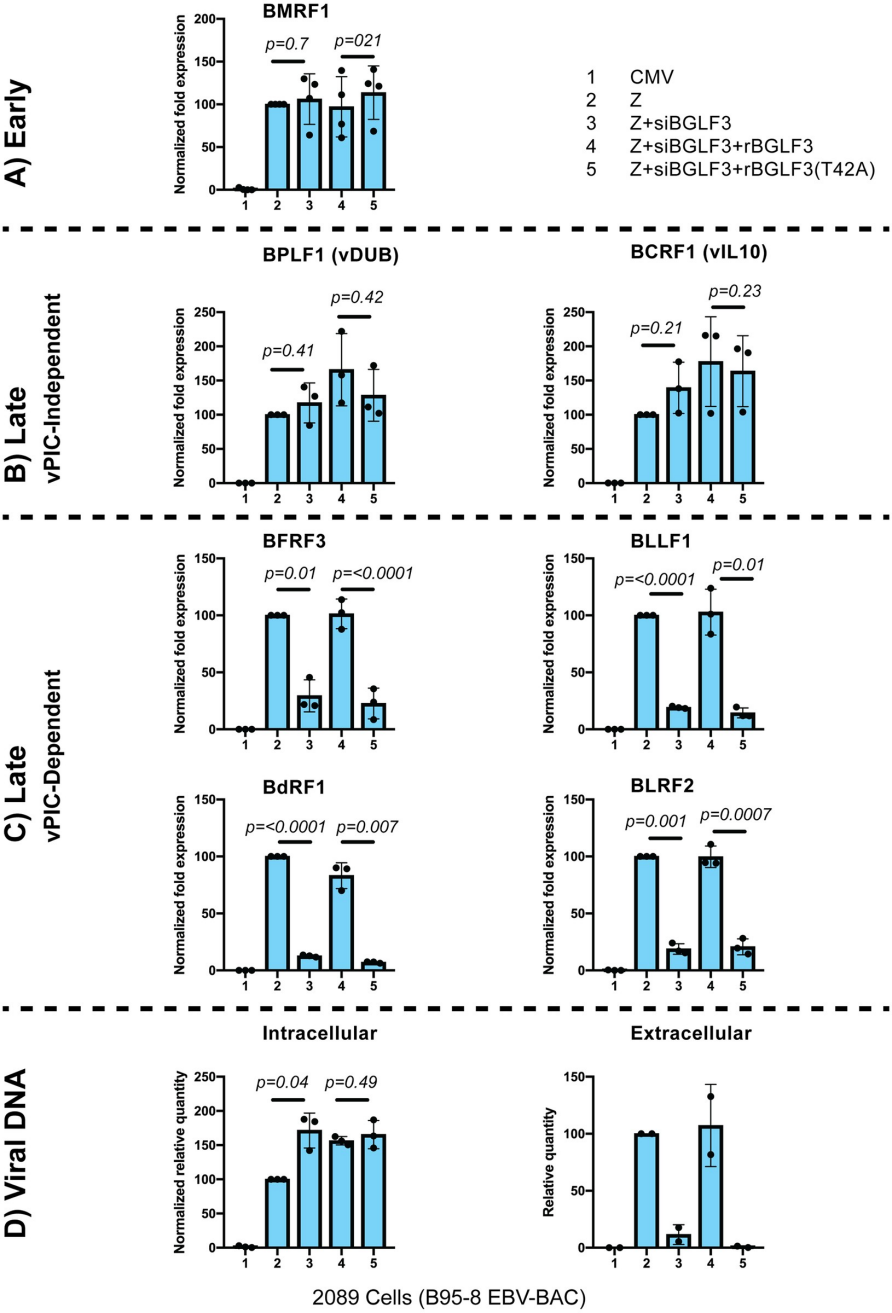
Mutation of threonine 42 in BGLF3 selectively abolishes transcription of late genes. Comparison of the expression of EBV lytic genes in 2089 cells expressing wild type BGLF3 or the BGLF3(T42A) mutant. RT-qPCR was used to assess the abundance of seven EBV lytic transcripts, these are: (A) early BMRF1, (B) vPIC independent late BPLF1 and BCRF1, and (C) vPIC-dependent late BFRF3, BLLF1, BdRF1, and BLRF2. (D) the amount of intracellular and extracellular viral DNA. CMV, control empty vector; Z, ZEBRA; siBGLF3, siRNA to BGLF3; rBGLF3, siBGLF3-resistant BGLF3, and rBGLF3(T42A), siBGLF3-resistant BGLF3(T42A). Each dot represents a biological replicate.

To study the effect of abolishing phosphorylation of BGLF3 at threonine 42 on synthesis of late transcripts, we used RT-qPCR to assess the level of seven EBV lytic transcripts representing three different groups of viral lytic genes: (A) early transcript: BMRF1 (polymerase associated factor); (B) vPIC-independent late transcripts: BCRF1 (viral IL10) and BPLF1(viral deubiquitinase), and (C) vPIC-dependent late transcripts: BFRF3 (capsid), BLLF1 (glycoprotein), BdRF1 (scaffold), and BLRF2 (tegument). We found that expression of all seven lytic genes was up-regulated in samples transfected with the lytic cycle activator, ZEBRA, relative to cells transfected with empty vector (CMV) (Fig 4A, 4B and 4C columns 1 and 2). Co-transfection of siBGLF3 significantly reduced the level of the four vPIC-dependent late transcripts, BFRF3, BLLF1, BdRF1, and BLRF2 but did not affect the level of early or vPIC-independent late transcripts (Fig 4A, 4B and 4C column 3). Expression of rBGLF3 suppressed the effect of siBGLF3 and restored expression of the four BGLF3-dependent late genes (Fig 4A, 4B and 4C column 4). However, alanine substitution of threonine 42 disrupted the capacity of rBGLF3 to support expression of the four BGLF3-dependent late genes (Fig 4A, 4B and 4C column 5).

To determine whether mutating threonine 42 to alanine affects the process of viral DNA amplification, we purified DNA from aliquots of the same cells that were examined in Figs 2A, 4A, 4B and 4C for protein and RNA expression, respectively. We found that expression of ZEBRA increased the level of viral DNA replication by an average of 100-fold relative to empty vector (CMV) (Fig 4D—Intracellular). Knockdown of BGLF3 or mutating the phospho-receptor threonine residue (T42) to alanine did not compromise the extent of viral genome amplification (Fig 4D—Intracellular). However, alanine substitution of phospho-T42 annihilated the capacity of the virus to produce new virus particles, as assessed by detecting the amount of the extracellular viral DNA (Fig 4D—Extracellular). These findings show that phosphorylation of BGLF3 at threonine 42 is essential for the capacity of the protein to support transcription of late genes encoding EBV structural proteins and hence production of new virions but is dispensable for viral DNA replication.

### Ectopic expression of vPIC components partially suppresses the phenotype of BGLF3(T42A)

As a hub protein, the function of BGLF3 in transcription of late genes is likely to be influenced by the protein’s capacity to interact with other subunits of the viral pre-initiation complex. A conceivable explanation for the phenotype of BGLF3(T42A) is that lack of phosphorylation at T42 might compromise the ability of the mutant BGLF3 protein to interact with one or more components of vPIC. Previous work studying assembly of various protein complexes demonstrated that point mutations that reduce the affinity of a protein to a complex could be overcome by increasing the concentration of the protein’s respective interactors in the complex [27, 28].

**Fig 5:**
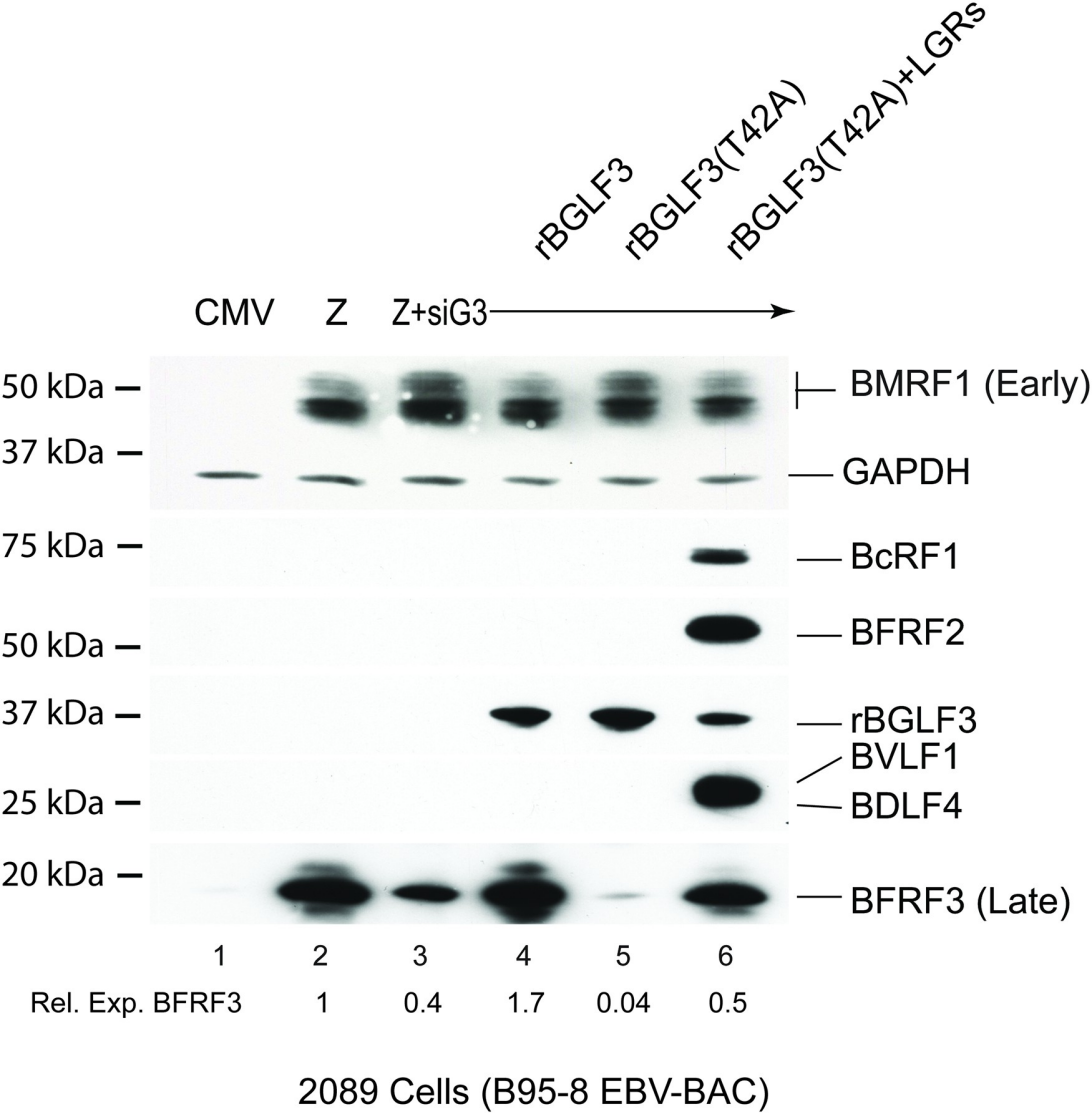
vPIC partially overcomes the defect in the capacity of BGLF3(T42A) to activate late gene expression. Western blot analysis of 2089 cell lysates demonstrating the capacity of vPIC to suppress the phenotype of BGLF3(T42A) and partially restore expression of late genes. Cells were transfected with empty vector (CMV), ZEBRA (Z), and ZEBRA plus siBGLF3. Knockdown of endogenous BGLF3 was complemented with siBGLF3-resistant wild type BGLF3 (rBGLF3) or mutant protein [rBGLF3(T42A)] in the absence and presence of four late gene regulators (LGRs): BcRF1, BDLF4, BFRF2, and BVLF1. Cell lysates were prepared 48h after transfection and were analyzed by immunoblotting. All late gene regulators were detected with anti-FLAG antibody. GAPDH served as a loading control. The membrane was re-blotted with antibody specific to early BMRF1 and late BFRF3 proteins. The experiment is a representative of three biological replicates.

To test the postulate that increasing the concentration of vPIC proteins might suppress the defect in BGLF3(T42A) and restore late gene expression, we eliminated expression of endogenous BGLF3 in 2089 cells using siRNA. Absence of endogenous BGLF3 was complemented by ectopic expression of wild type rBGLF3 or rBGLF3(T42A). Similar to Fig 2, expression of rBGLF3(T42A) failed to support synthesis of the late BFRF3 protein. Co-expression of four late gene regulators (BcRF1, BDLF4, BFRF2, and BVLF1) partially suppressed the phenotype of rBGLF3(T42A) and increased the expression level of BFRF3 to 54% relative to cells transfected with ZEBRA alone (Fig 5 compare lanes 2 and 6). This result suggests that increasing the protein concentration of four late gene regulators can partially suppress the defect in BGLF3(T42A) and restore expression of late genes.

### BFRF2 and BVLF1 are the only two components of vPIC that are essential to partially suppress the phenotype of BGLF3(T42A)

**Fig 6:**
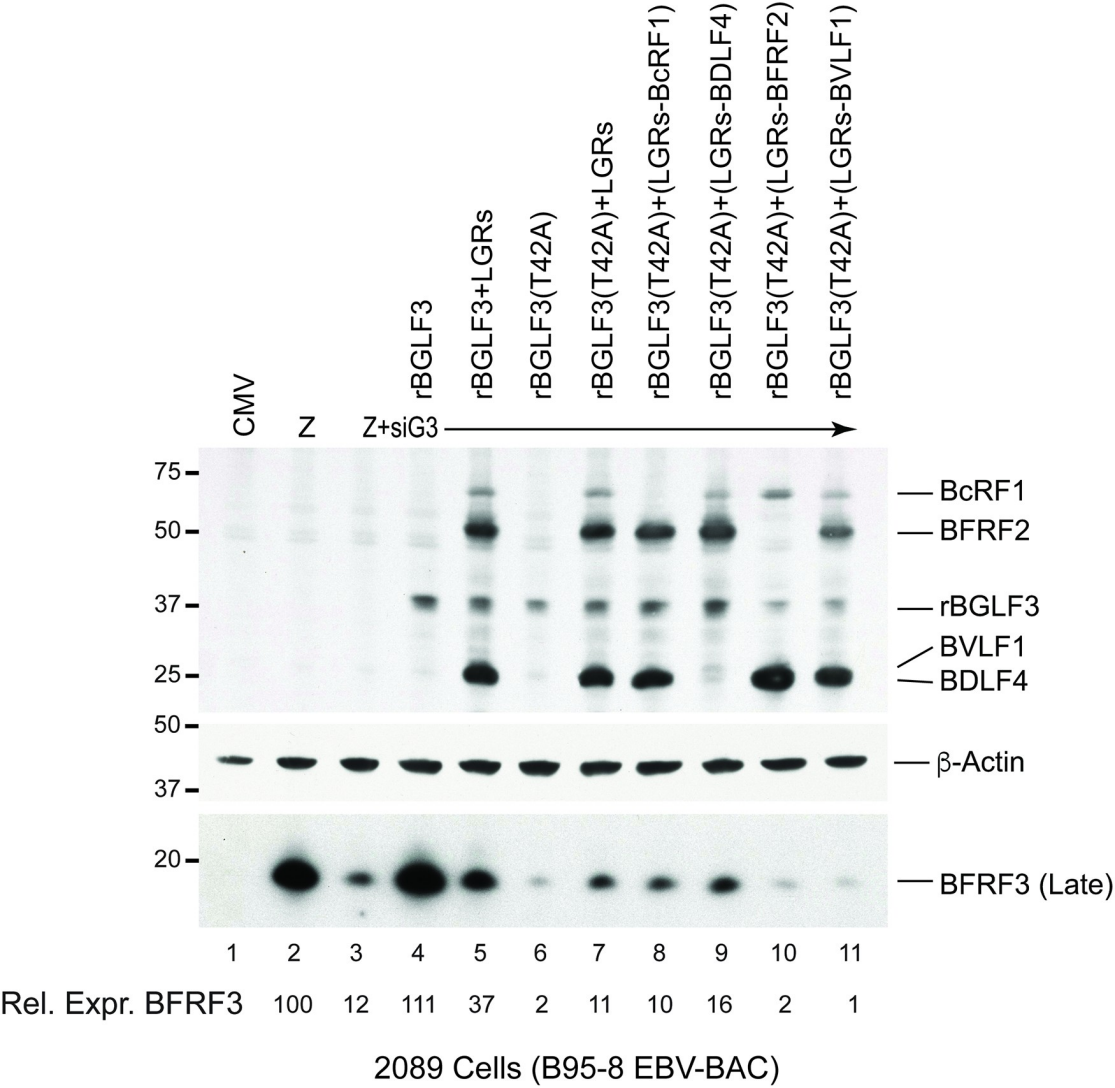
Elimination of BFRF2 or BVLF1 abolishes the capacity of vPIC to suppress the late gene expression defect of BGLF3(T42A). Western blot analysis of 2089 cells transfected with empty vector (CMV), ZEBRA (Z), and ZEBRA plus siBGLF3 (siG3). Knockdown of endogenous BGLF3 was complemented with siBGLF3-resistant wild type BGLF3 (rBGLF3) or mutant protein [rBGLF3(T42A)]. Failure of BGLF3(T42A) to activate expression of the late BFRF3 protein was partially overcome by expressing a mixture of four vPIC proteins (BcRF1, BDLF4, BFRF2, and BVLF1). Components of vPIC were individually omitted from the complex to assess the role each late gene regulator plays in suppressing the phenotype of BGLF3(T42A). The membrane was blotted with antibodies specific to β-actin (gel loading control), late BFRF3 and FLAG epitope for detection of late gene regulators. The experiment is a representative of two biological replicates.

To delineate the contribution of each late gene regulator in restoring expression of EBV structural proteins, we expressed BGLF3(T42A) in 2089 cells together with different mixtures of vPIC components. In each mixture, one of the four late gene regulators was omitted. Cells were harvested after 48 hours and protein lysates were prepared and analyzed by Western blotting to assess the level of the late BFRF3 protein. We found that eliminating BFRF2 or BVLF1 from the mixture of late gene regulators abolished the ability of vPIC to restore expression of the late BFRF3 protein; however, omission of BcRF1 and BDLF4 had no effect (Fig 6).

### BFRF2 and BVLF1 are sufficient to partially rescue the defect in BGLF3(T42A)

**Fig 7:**
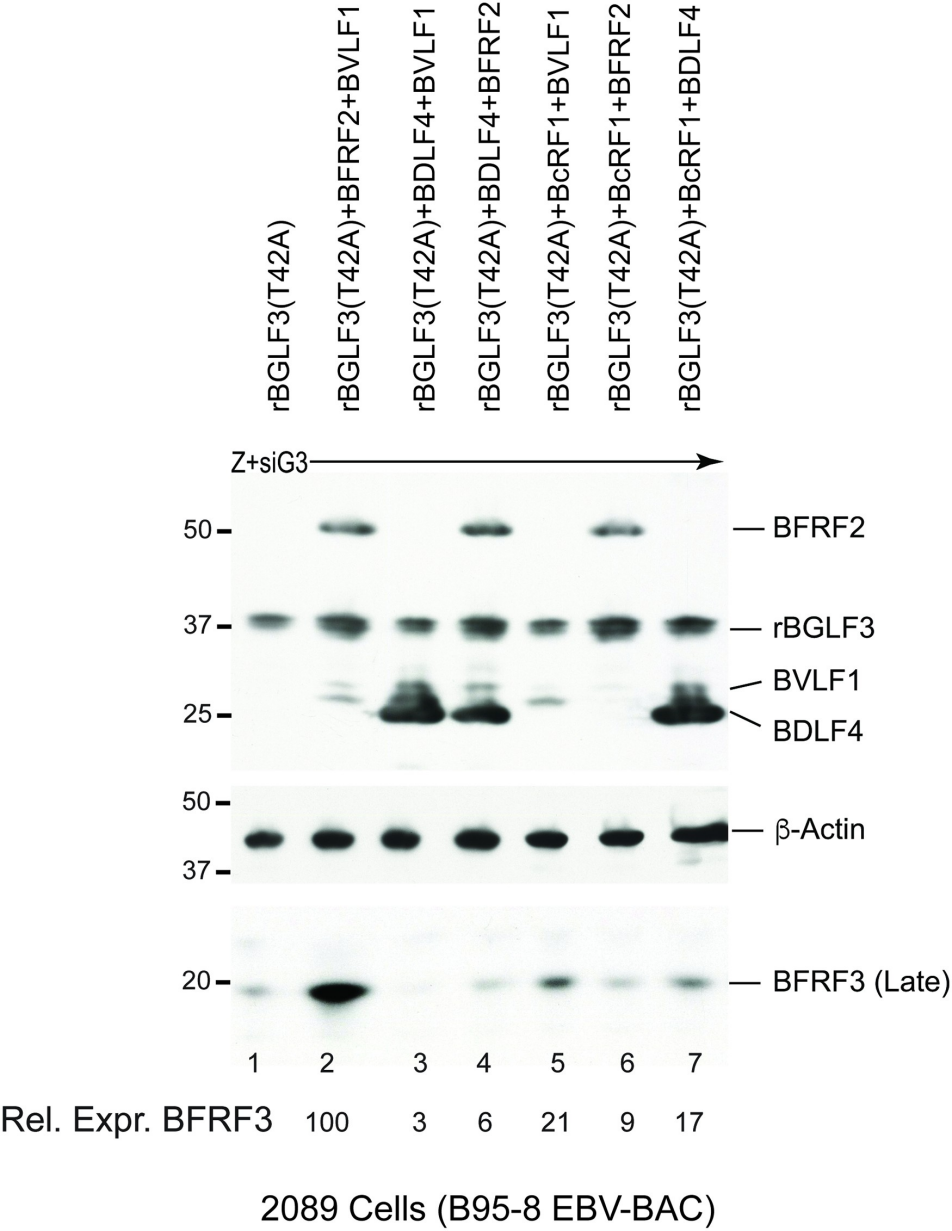
Co-expression of BFRF2 and BVLF1 is sufficient to restore synthesis of late proteins in the presence of BGLF3(T42A). Western blot analysis of 2089 cell lysates demonstrating the ability of BFRF2 and BVLF1 to overcome the BGLF3(T42A) defect in activating late gene expression. Cells were transfected with ZEBRA plus siBGLF3 to knockdown expression of endogenous BGLF3. Lack of endogenous BGLF3 was supplemented by expression of rBGLF3(T42A). Different combinations of the various components of vPIC were co-expressed to restore late gene expression. All late gene regulators were detected with anti-FLAG antibody. β-actin served as a loading control. The membrane was re-blotted with antibody specific to late BFRF3 protein. The capacity of BFRF2 and BVLF1 to suppress the phenotype of BGLF3(T42A) by partially restoring expression of the late BFRF3 protein was reproducible in three biological replicates.

Since BFRF2 and BVLF1 are the only two proteins necessary for the ability of vPIC to restore synthesis of late products in cells expressing BGLF3(T42A), we asked whether provision of these two proteins was sufficient to suppress the defect in BGLF3(T42A). We transfected 2089 cells with ZEBRA to induce the lytic cycle. Expression of endogenous BGLF3 was knocked down using siBGLF3. Lack of BGLF3 was complemented with the mutant rBGLF3(T42A). We found that co-transfection of BFRF2 and BVLF1 suppressed the phenotype of the T42 mutation and partially restored synthesis of the late BFRF3 protein (Fig 7, lane 2). To assess the specificity of the BFRF2/BVLF1 combination, we studied the capacity of all possible combinations of the four late gene regulators to suppress the phenotype of rBGLF3(T42A). BFRF2/BVLF1 was the most competent combination to restore late gene expression in 2089 cells complemented with rBGLF3(T42A) (Fig 7). Our findings indicated a novel functional interaction between the phosphoacceptor threonine 42 of BGLF3 and the two late gene regulators, BFRF2 and BVLF1.

In summary, lack of phosphorylation at T42 has detrimental effects on synthesis of EBV structural proteins; however, this defect could be partially complemented by increasing the concentration of BFRF2 and BVLF1.

### BGLF3 and BGLF3(T42A) are competent to interact with BcRF1

Based on previous protein interaction studies, BGLF3 serves as a bridge connecting BcRF1, the component of vPIC that binds to late promoters, to other subunits of the complex. To determine whether phosphorylation of BGLF3 at threonine 42 mediates the protein’s capacity to interact with BcRF1, we compared interaction of wild type BGLF3 or BGLF3(T42A) with BcRF1. Co-immunoprecipitation was performed using 2089 cells expressing either form of FLAG-tagged BGLF3 in the absence and presence of BcRF1. We found that both BGLF3 and BGLF3(T42A) interacted with BcRF1 to the same extent (S1 Fig lanes 3 and 5). This result suggests that wild type and mutant BGLF3 could be equally recruited to late promoters via their interaction with BcRF1. Furthermore, it corroborates our findings in Figs 6 and 7 demonstrating that ectopic expression of BFRF2 and BVLF1, but not BcRF1, was essential to partially suppress the phenotype of BGLF3(T42A) and restore expression of late genes.

### BGLF3(T42A) is defective in interacting with the BFRF2:BVLF1 subcomplex

**Fig 8:**
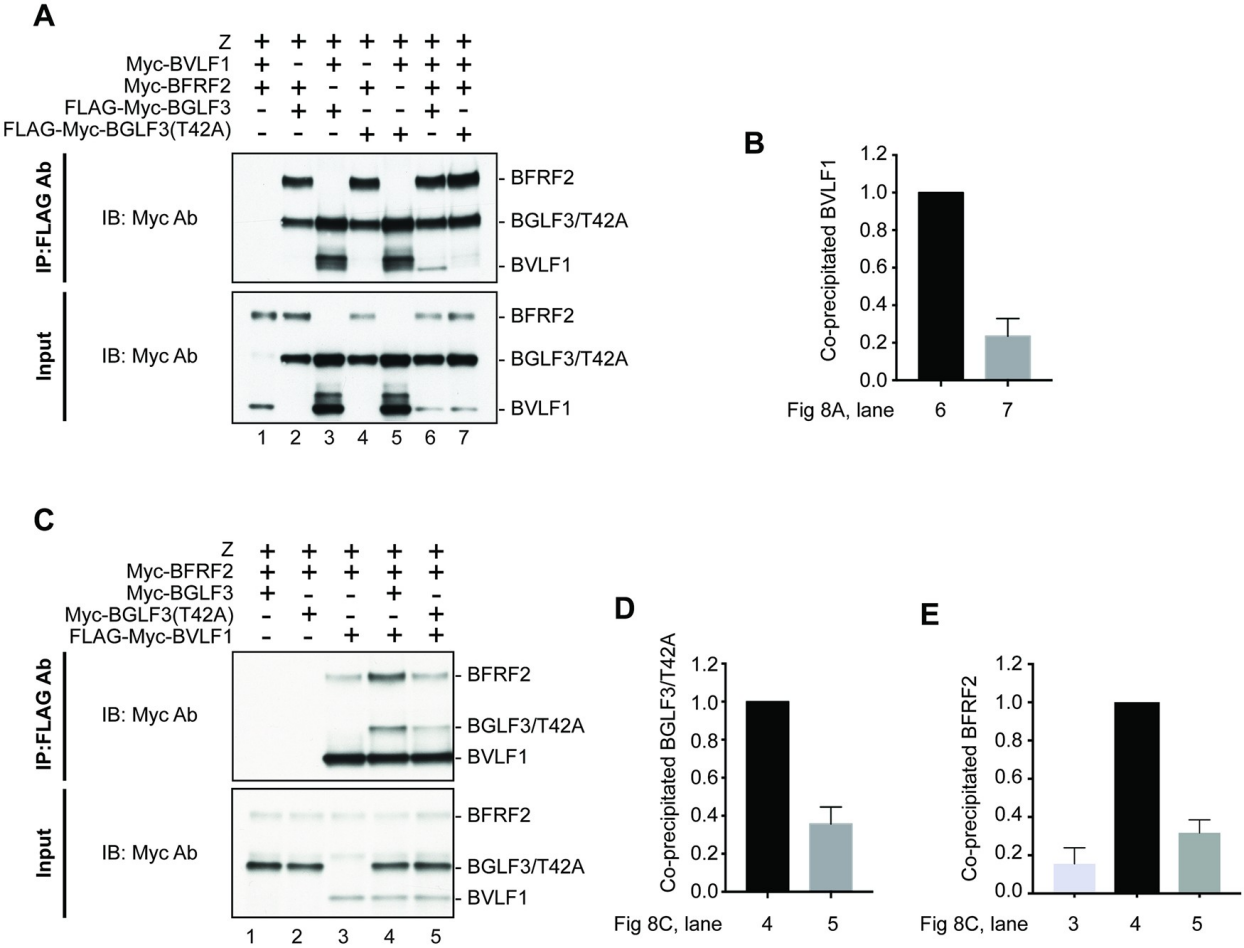
Phosphoacceptor threonine 42 of BGLF3 is necessary for BGLF3, BFRF2 and BVLF1 to form a trimeric complex. Western blot analysis assessing the capacity of BGLF3 to interact with BFRF2 and BVLF1 in the absence and presence of the phosphoacceptor threonine 42 residue. 2089 cells were transfected with the indicated vectors and harvested after 48h. Cleared cell lysates were subjected to immunoprecipitation using anti-FLAG affinity gel followed by Western blot analysis to detect the ability of BGLF3 and BGLF3(T42A) to interact with BFRF2 and BVLF1 individually or together. (A) Immunoprecipitation using FLAG-tagged BGLF3. (B) Quantitation comparing the level of BVLF1 protein co-immunoprecipitated with wild type (lane 6) or mutant (lane 7) BGLF3 in the presence of BFRF2. The level of BVLF1 was corrected to BGLF3 and BGLF3(T42A), respectively. (C) Co-immunoprecipitation of BFRF2 and BGLF3 proteins using FLAG-tagged BVLF1. (D) and (E) quantitation of the level of BGLF3 and BFRF2 proteins that co-precipitate with BVLF1, respectively. Input samples represent 5% of cleared cell lysates. Quantitation is based on two independent experiments. Membranes were blotted with anti-Myc antibody for detection of BFRF2, BGLF3, and BVLF1.

To understand the nature of the functional interaction between phospho-threonine 42 in BGLF3 and the BFRF2 and BVLF1 proteins, we used co-immunoprecipitation to study the potential protein complexes formed by these three proteins. In Fig 8A, we assessed the capacity of BGLF3 and BGLF3(T42A) to interact with BFRF2 and BVLF1 when expressed individually or together in transfected 2089 cells. Neither BFRF2 nor BVLF1 were non-specifically immunoprecipitated in the absence of FLAG-tagged BGLF3 (Fig 8A lane 1). Both wild type BGLF3 and the BGLF3(T42A) mutant interacted with BFRF2 and BVLF1 when provided individually in a pairwise co-immunoprecipitation (Fig 8A lanes 2, 3, 4, and 5). Co-expression of BFRF2 and BVLF1 in the presence of wild type BGLF3 resulted in a trimeric complex (Fig 8A lane 6). However, interestingly, mutation of threonine 42 abolished the interaction between BGLF3 and BVLF1 without affecting the interaction between BGLF3 and BFRF2 (Fig 8A lane 7).

To further confirm formation of a trimeric complex that includes BGLF3, BFRF2, and BVLF1, we performed reciprocal co-immunoprecipitation using FLAG-tagged BVLF1 to pull down BFRF2 alone or together with BGLF3. In the absence of BGLF3, BVLF1 had weak affinity to the BFRF2 protein (Fig 8B). However, interaction of BVLF1 and BFRF2 increased substantially in the presence of wild type BGLF3, around 6-fold relative to no BGLF3 based on two independent experiments (Fig 8E). Mutation of threonine 42 to alanine compromised the ability of BGLF3 to form a stable complex with BFRF2 and BVLF1 (Fig 8D and 8E). Our experiments demonstrating that either BGLF3 or BVLF1 can pull down the other two components of the subcomplex suggest that all three proteins assemble into a trimeric complex. Abolishing phosphorylation of BGLF3 at T42, disrupts formation of this trimeric complex and functionally impairs expression of EBV transcripts encoding structural proteins (Fig 4).

## Discussion

**Fig 9:**
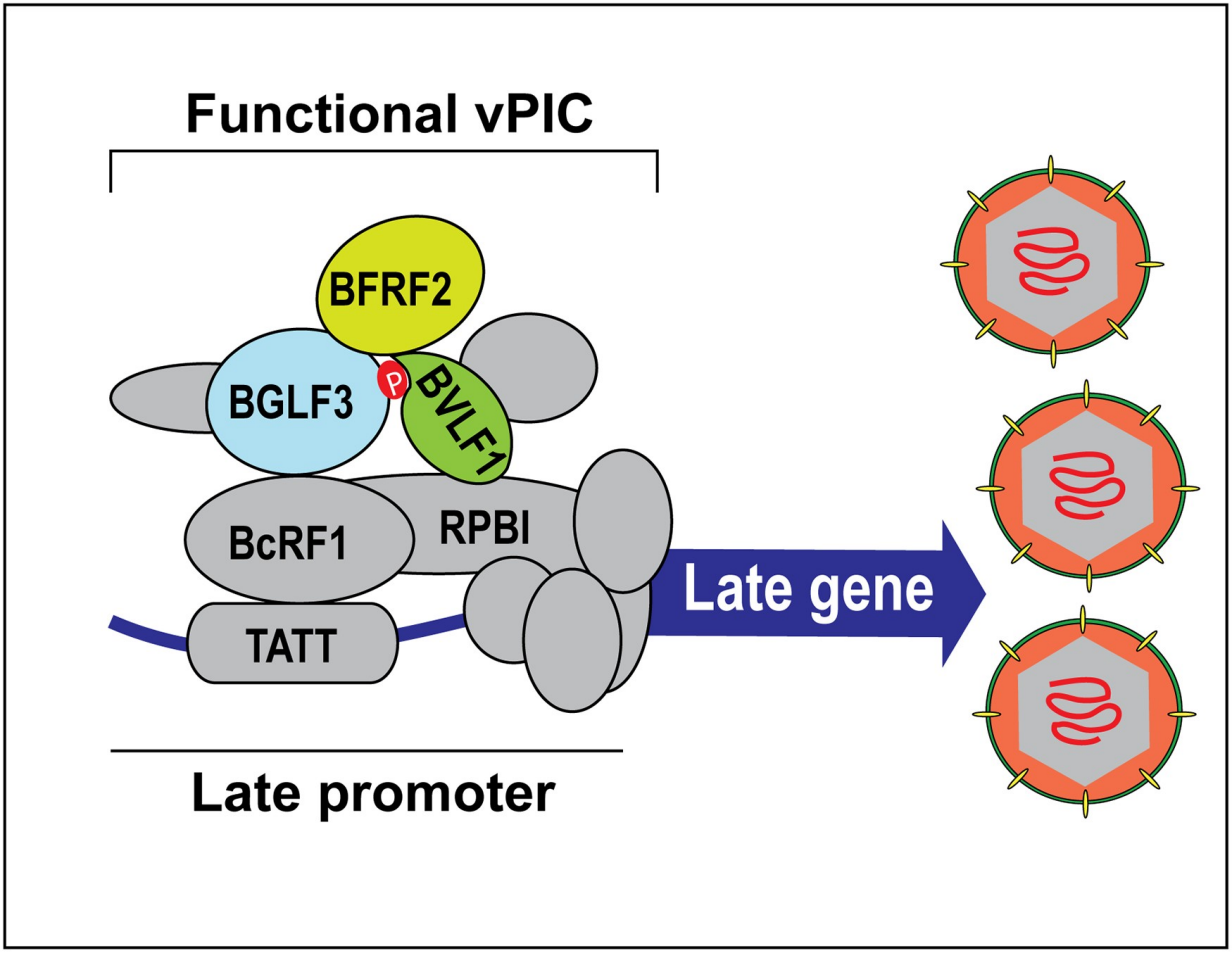
Model depicting the role of phosphorylation of BGLF3 at T42 in assembly of vPIC. BGLF3 is a core component of EBV transcription pre-initiation complex (vPIC). BGLF3 is phosphorylated at T42, a site conserved in gammaherpesviruses. Phosphorylation of BGLF3 at T42 is essential for recruitment of BVLF1 to the preinitiation complex. Lack of phosphorylation at T42 abolishes expression of EBV structural proteins and production of virus particles.

Phosphorylation represents one of the most important post-translational modifications that regulates the activity, interaction, localization, and stability of proteins. Recently, we and others identified a viral pre-initiation complex dedicated to transcription of beta and gamma herpesvirus late genes. Previous studies mapped a number of important protein interactions that contribute to our current understanding of the overall organization of vPIC. However, none of these studies addressed the role of post-translational modifications, particularly phosphorylation, in regulating assembly or function of vPIC in transcription of late genes. Here, we studied phosphorylation of BGLF3, a protein that is indispensable for transcription of late genes and serves as a core protein connecting BcRF1, the vTBP, to other components of the vPIC module. We report the following novel findings: 1) BGLF3 is phosphorylated *in vivo* at threonine 42, a site conserved in other gamma herpesviruses, during the late phase of lytic infection. 2) Phosphorylation of BGLF3 at T42 is crucial for the role of the protein in transcription of late genes. 3) BFRF2 and BVLF1 suppress the defect observed in transcription of late genes due to lack of phosphorylation of BGLF3 at threonine 42. 4) As a result of phosphorylation of BGLF3 at threonine 42, a novel trimeric subcomplex forms that includes BFRF2, BGLF3 and BVLF1. 5) Phosphorylation of BGLF3 does not regulate its interaction with BcRF1 (S1 Fig) suggesting that lack of phosphorylation does not impact recruitment of BGLF3 to late promoters but abolishes binding of BVLF1 to BGLF3 and hence formation of a functional vPIC. Our findings demonstrate that phosphorylation at T42 is crucial for BGLF3 to interact functionally and physically with BFRF2 and BVLF1 (Fig 9). These findings demonstrate that post-translational modifications regulate the function of vPIC in transcription of late genes.

### Formation of subcomplexes: An alternative model for assembly of vPIC

Much of the current understanding of the overall organization of vPIC is derived from previous studies using pairwise co-immunoprecipitation experiments. These studies suggest that components of vPIC are involved in an intricate network of protein-protein interactions that form a functional pre-initiation complex. While these studies present a plausible model for the overall structure of the complex, several questions remain unanswered. For instance, what are the dynamics of vPIC assembly? Do these mapped protein interactions take place simultaneously to generate one main complex, as previously proposed, or does assembly of vPIC occur in a dynamic stepwise manner that involves formation of various subcomplexes of late gene regulators? Formation of these subcomplexes might be strictly regulated by certain post-translational modifications to synchronize the proper assembly of vPIC and the timing at which a particular late gene regulator is added or removed from the complex. It is also conceivable that a late gene regulator, such as BGLF3, might be involved in more than one subcomplex. This interpretation might explain how BGLF3 accommodates multiple interactions previously reported using pairwise coimmunoprecipitation. Furthermore, the time at which a particular late gene regulator is added to the complex is also significant. The BVLF1 protein, for example, might be recruited to the complex at a later time point. UL79, the CMV ortholog of EBV BVLF1, was previously shown to promote the transcriptional elongation activity of RNAPII [25]. This result posits the question of whether BVLF1 is part of the viral pre-initiation complex during promoter recognition or the protein is recruited to the complex as RNAPII exits the promoter.

### Formation of the trimeric subcomplex BFRF2:BGLF3:BVLF1 is crucial for expression of late genes

Our data demonstrate that BGLF3 is phosphorylated at threonine 42 (Fig 1). Mutation of this phopshoacceptor residue abolished expression of the late BFRF3 protein and markedly reduced transcription of several late transcripts (Figs 2 and 4). The effect of mutating T42 to alanine was selective to vPIC-dependent late genes encoding structural proteins; expression of early genes or vPIC-independent late genes was not affected (Fig 4). As a core protein, BGLF3 coordinates multiple interactions within vPIC. Failure to synthesize late transcripts suggested a defect in the capacity of the BGLF3 mutant to establish a specific interaction with one or more late gene regulators that form vPIC. Previous reports demonstrated that point mutations that reduce the affinity of a protein to a particular complex could be suppressed by increasing the concentration of the protein’s respective partners in the complex [27, 28]. Following a similar approach, we managed to partially suppress the phenotype of BGLF3(T42A) and restore expression of late genes by increasing the protein concentration of two specific components of vPIC, BFRF2, and BVLF1 (Figs 6 and 7). The combined effect of BFRF2 and BVLF1 was not reproduced when other combinations of late gene regulators were ectopically expressed (Fig 7). These findings led to the hypothesis that phospho-threonine 42 is likely to mediate or regulate interaction of BGLF3 with BFRF2 and/or BVLF1. Indeed, co-immunoprecipitation experiments demonstrated that BFRF2, BVLF1, and the phosphorylated form of BGLF3 interact together. The ability of BGLF3 and BVLF1 to co-precipitate the two other proteins suggests that BFRF2, BGLF3, and BVLF1 form a stable trimeric subcomplex of that is essential for the assembly of a functional vPIC (Fig 8). Phosphorylation of BGLF3 at threonine 42 is likely to augment the affinity of BVLF1 to the BFRF2:BGLF3 subcomplex. In our experiments, removal of phospho-threonine 42 weakens binding, while higher levels of the BVLF1 and BFRF2 proteins partially restore interaction with BGLF3(T42A) by slightly shifting the equilibrium towards complex formation (Fig 8C). Our data suggest that formation of this trimeric subcomplex is essential for transcription of late genes. Alanine mutation of BGLF3 at T42 disrupted complex formation (Fig 8) and markedly reduced expression of vPIC-dependent late genes (Figs 2 and 4). Furthermore, failure of BFRF2 and BVLF1 to fully restore expression of late genes (Fig 7) is corroborated by the reduced ability of the BGLF3(T42A):BFRF2 subcomplex to interact with BVLF1 (Fig 8C). Collectively, our approach involving mutation of BGLF3 at threonine 42 and suppression of the BGLF3(T42A) phenotype strongly correlates with our protein interaction studies to demonstrate the importance of the BFRF2:BGLF3:BVLF1 trimeric subcomplex in transcription of late genes (Fig 9).

### Formation of the trimeric complex BFRF2:BGLF3:BVLF1 is dependent on phosphorylation of T42

In our efforts to understand the phenotype of BGLF3(T42A) we compared the ability of wild type and mutant BGLF3 proteins to interact with BVLF1. Using pairwise immunoprecipitation experiments, both wild type BGLF3 and BGLF3(T42A) were equally competent to interact with BVLF1 (Fig 8A lanes 3 and 5). Addition of BFRF2 to the complex revealed a substantial defect in the ability of BGLF3(T42A) to bind to BVLF1. One possible explanation of this outcome is that association of BFRF2 with BGLF3 results in a new interface that accommodates interaction with BVLF1 in a manner dependent on phosphorylation of BGLF3 at threonine 42. BFRF2 and BVLF1 might form a pocket that fits the motif encompassing phospho-T42 in the BGLF3 protein. Our data suggests that phosphorylation regulates formation of this trimeric complex but is not involved in recruitment of BGLF3 to late promoters; both wild type BGLF3 and BGLF3(T42A) are capable of interacting with BcRF1, the vTBP-like protein that recognizes late promoters (S1 Fig). Therefore, lack of phosphorylation at T42 in BGLF3 is likely to impede subsequent binding of other subunits to form a functional pre-initiation complex.

One approach that is frequently used to study a constitutively phosphorylated site is to mutate this phosphoacceptor residue into a phosphomimetic one. In Fig 3A, we mutated T42 to aspartate, and glutamate residues. None of the phosphomimetic mutations restored expression of late genes; however, mutating T42 to a different phosphorylatable residue, serine, maintained expression of late genes (Fig 3B). This outcome is not unexpected considering a phosphoamino acid has unique chemical characteristics relative to other amino acids including aspartate and glutamate. A phosphate group in a phosphoamino acid has a bigger hydrated shell and more negative charge relative to a carboxyl group in a phosphomimetic residue [29]. Furthermore, a phosphate group forms stronger and more stable hydrogen bonds and salt bridges in protein-protein interactions relative to a carboxyl group [30]. Signal transducing adaptor proteins, such as 14-3-3 protein and proteins containing FHA- or SH2-domains, are phospho-binding proteins that are incapable of recognizing a phosphomimetic replacement [31–33]. Studying the structure of these interactions revealed that phosphomimetic residues do not fit in the binding pocket of adaptor proteins [31, 34, 35]. Our results suggest that transcription of late genes is dependent on the presence of a phosphate group at position 42.

A phosphomimetic mutation is also less likely to substitute for the presence of a phosphate group if both phosphorylated and non-phosphorylated forms play distinct roles in the function of the protein. It is conceivable that both phosphorylated and non-phosphorylated forms of BGLF3 play separate roles in transcription of late genes. BGLF3 is capable of interacting with BVLF1 and BFRF2 individually, such interaction might occur at a specific stage of transcription of late genes that differs from that requiring formation of the trimeric complex.

An alternative interpretation of our data is that BGLF3 has two separate motifs for interaction with BVLF1. One motif binds to nonmodified BVLF1 and a second BGLF3 motif that binds to modified BVLF1. In the absence of BFRF2, BGLF3 favors interaction with the modified form of BVLF1. However, in the presence of BFRF2, BGLF3 interacts with the nonmodified form of BVLF1. Interaction of BGLF3 with the nonmodified form of BVLF1 is dependent on phosphorylation of BGLF3 at threonine 42. Changes in modification of BLVF1 and its impact on formation of various subcomplexes might represent different stages during the process of transcription of late genes. We are currently studying the possibility that BVLF1 is modified and assessing its role in transcription of late genes.

In conclusion, our results demonstrate the essential role protein phosphorylation plays in regulating the function of vPIC during transcription of late genes. Lack of a single phosphorylation site in BGLF3 abolishes expression of late structural proteins and prevents virus release. Phosphorylation of late gene regulators might serve as checkpoints to ensure the precise timing for assembly of vPIC subcomplexes during synthesis of late products (Fig 9). Identifying additional post-translational modifications that are indispensable for expression of late genes and the responsible modifying enzymes has the potential to inform the generation of a novel class of drugs against EBV and its associated diseases.

## Materials and methods

### Expression vectors and antibodies

The ZEBRA protein expression vector was constructed as previously described [36]. The constructs expressing BGLF3, BcRF1, BFRF2, BVLF1, and BDLF4 were cloned into the eukaryotic pCMV6-Entry vectors using the SfgI and MluI restriction sites. The mutants BGLF3(T42A), BGLF3(T42D), BGLF3(T42E), and BGLF3(T42S) were generated by introduction of the indicated point mutations in the BGLF3 sequence using the following mutagenic primers: 5´-CAGTTTAAGCTCGTGGAGGCGCCCCTGAAGTCCTTTC-3’, 5’- CAGTTTAAGCTCGTGGAGGACCCCCTGAAGTCCTTTC-3´, 5’-CAGTTTAAGCTCGTGGAGGAGCCCCTGAAGTCCTTTC-3’, and 5’- AACAGTTTAAGCTCGTGGAGTCGCCCCTGAAGTCCTTTCTG-3’ and their complementary strands, respectively. siRNA-resistant BGLF3 (rBGLF3) was produced by inserting silent mutations in the region of the late gene regulator mRNA that is recognized by the siRNA. These silent mutations disrupt the complementarity between the siRNA and BGLF3 mRNA without affecting the amino acid sequence of the protein. Production of rBGLF3 and experiments establishing specificity of the utilized BGLF3 siRNA were described in details in our previous studies [18, 19]. The following commercial antibodies were used in Western blotting: monoclonal anti-FLAG M2 antibody (Sigma); Anti-Myc-Tag rabbit monoclonal antibody (Cell signaling); anti-β-Actin antibody (Sigma); anti-GAPDH antibody (abcam). Antibodies to BMRF1 (EAD), ZEBRA, and BFRF3 were previously described [37, 38].

### Cell culture and transfection

2089 cells are human embryonic kidney (HEK) 293 cells stably transfected with a bacmid containing wild-type EBV B95-8 genome [39, 40]. Cells were cultured in Dulbecco’s modified Eagle medium (DMEM) supplemented with 10% fetal bovine serum (FBS) (Gibco), and penicillin-streptomycin at 50 units/ml. Hygromycin B (Calbiochem) 100 μg/ml was added to the medium to select for 293 cells containing the EBV bacmid. The HH514-16 Burkitt lymphoma cell line is a subclone of EBV-infected P3J-HR-1 cell line [41]. SNU-719 cells is a gastric carcinoma cell line derived from a human tumor biopsy naturally infected with EBV [42]. The eukaryotic plasmids were transfected using lipofectamine 2000 (Invitrogen) following the manufacture’s protocol. Transfections were carried out in OPTI-MEM medium (Gibco). Cells were incubated at 37° C in 5% CO_2_ incubator and harvested 48 h after transfection.

### Immunoblotting

Harvested cells were lysed in sodium dodecyl sulfate (SDS) sample buffer at 10^6^ cell/10ul. After sonication, protein lysates were denatured at 100°C for 5 min and resolved on 10% SDS-polyacrylamide gel or 4–15% Criterion TGX Precast Protein Gel (Bio-Rad). Resolved proteins were transferred to a nitrocellulose membrane (Bio-Rad). The membrane was blocked in TBS buffer (50 mM Tris-Cl, pH 7.5 and 150 mM NaCl) supplemented with 5% non-fat milk and 0.1% Tween-20. Nitrocellulose membranes were blotted with specific primary antibodies to cellular and viral proteins. Immunocomplexes were visualized by ECL (GE) or by autoradiography using ^125^I-protein A (PerkinElmer).

### Immunoprecipitation

2089 cells were harvested, washed in cold phosphate-buffered saline, and resuspended in lysis buffer (20 mM Tris-HCl, pH 7.5, 150 mM NaCl, 1 mM Na2EDTA, 1 mM EGTA, 1% Triton) containing Halt Protease and Phosphatase inhibitors(ThermoFisher). Lysates were passed through a 25-G needle 9 times then centrifuged at 21,000 x *g* for 10 min at 4°C. Supernatents were pre-incubated with protein A agarose beads to reduce non-specific interactions. Five percent of each supernatant was stored at -80°C as input sample. The rest of the supernatant was incubated with pre-washed anti-FLAG M2 affinity agarose beads (Sigma) for 2h at 4°C. The beads were washed four times with lysis buffer and once with elution buffer (50 mM HEPES, pH 7.4, 100mM NaCl, 1 mM DTT, 5 mM βglycerophosphate, 0.1 mM Na_3_VO_4_, 0.01% Igepal CA630, 10% glycerol). Immunoprecipitated proteins were eluted in elution buffer containing 0.5mg/ml 3X FLAG Peptide (Sigma). Input samples and immunoprecipitated proteins were detected by Western blotting using appropriate antibodies or by protein staining using colloidal Coomassie blue [43].

### Phospho-enrichment and LC MS/MS analysis

Sample preparation of the liquid chromatography-tandem mass spectrometry (LC MS/MS) analysis was performed following the previously described protocol [44]. Briefly, immunoprecipitated proteins were subjected to Dithiothreitol (DTT) reduction, Iodoacetamide (IAN)-mediated alkylation followed by trypsin digestion. The digested sample was desalted by Spin Desalting column (Thermo) and acidified with 0.5% Trifluoroacetic acid (TFA), 50% acetonitrile then subjected to titanium dioxide enrichment using the Top Tips system (Glygen Corp). The resulting phosphopeptide-enriched sample, dissolved in 70% formic acid and diluted with 0.1% TFA, was then subjected to LC-MS/MS analysis using the Orbitrap Fusion Mass Spectrometer that is equipped with a Waters nanoACQUITY UPLC system. A Waters Symmetry C18 180 μm x 20 mm trap column and a 1.7 μm, 75 μm x 250 mm nanoACQUITY UPLC column was utilized for online peptide separation. The acquired data was peak picked and searched using the Mascot Distiller and the Mascot search algorithm, respectively. Manual examination of the MS/MS spectra (as shown in Fig 1B) and the corresponding assigned fragment ions were conducted to verify the identified phosphopeptide.

### Quantitative RT-PCR

RNA was prepared from cells using the Qia-shredder and the RNeasy Plus products from Qiagen. The concentration of RNA in each sample was determined by measuring the optical density at 260 nm. The level of viral transcripts was assessed from 100 ng of total RNA using iScript One-Step RT-PCR with SYBR Green (Bio-Rad) in a total volume of 25 μl. The level of 18S RNA was measured to normalize for the total amount of RNA. Each sample was analyzed in triplicate; the fold change in expression was calculated using the ΔΔC_T_ formula implemented in the software of the CFX real-time PCR system (Bio-Rad). The efficiency of the primers used in RT-qPCR was determined against 10-fold increasing concentrations of viral DNA. The sequences of the primers are provided in Table 1.

**Table 1:** Sequences of primers used in real time PCR assays.

Gene	Forward Primer (5’-3’)	Reverse Primer (5’-3’)
BCRF1	CAG GCC CTG TCA GAA ATG AT	TCC TTT TTC CTG CAG CTT GT
BdRF1	GCT ATC AGG TAA CGC AGG AG	GTT GGT CTG AAG CAG TGT CA
BFRF3	GCC ATA GAC AAG AGG CAG AG	CGG AGG CTG CTA ATA GAT GA
BLLF1	TGC TGA TCC CAA TAC AAC GA	TGG AGA TGG ACT TGG TGT CA
BLRF2	CTC TGA AGC AAC AGG TCC TC	TCC GAA CCT TGT CTT CAA TC
BMRF1	CAA CAC CGC ACT GGA GAG	GCC TGC TTC ACT TTC TTG G
BPLF1	CGG GGT CAC AGA AGA AAC AT	GTC CTG AGA GGA GGC TTG TG
oriLyt	TCC TCT TTT TGG GGT CTC TG	CCC TCC TCC TCT CGT TAT CC

### Quantification and statistical analysis

The relative expression levels of target proteins were measured according to the density of bands from Western blot using densitometer machine (GS-800 Calibrated Densitometer, Bio-Rad). Expression of the late BFRF3 protein was calculated relative to the expression level of GAPDH or β-Actin. Statistical analysis for viral transcripts (Fig 4) was performed using paired t test available in GraphPad Prism software (La Jolla, CA, USA). A value of *p* < 0.05 was considered statistically significant.

## Supporting information

S1 Fig BGLF3(T42A) is competent to interact withBcRF1, the viral TBP-like protein. Comparing the capacity of wild type BGLF3 and BGLF3(T42A) to interact with BcRF1 during lytic infection. 2089 cells were transfected with FLAG-tagged BGLF3 or BGLF3(T42A) with and without the viral TATA box binding protein BcRF1. ZEBRA was co-transfected in all the cells to induce the lytic cycle. Co-immunoprecipitation was carried out using FLAG antibody crosslinked to agarose beads. BGLF3 and associated proteins were eluted using 3X FLAG peptide. Myc antibody was used to detect both BGLF3 and BcRF1. As shown in lanes 3 and 5, both wild type BGLF3 and BGLF3(T42A) interact with equal affinity to BcRF1.
(TIF)
