# Supplementary figures and images for: A single phosphoacceptor residue in BGLF3 is essential for transcription of Epstein-Barr virus late genes
Source: PLoS Pathog. 2019 Aug 28;15(8):e1007980. doi: 10.1371/journal.ppat.1007980 (PMC6713331; doi:10.1371/journal.ppat.1007980)

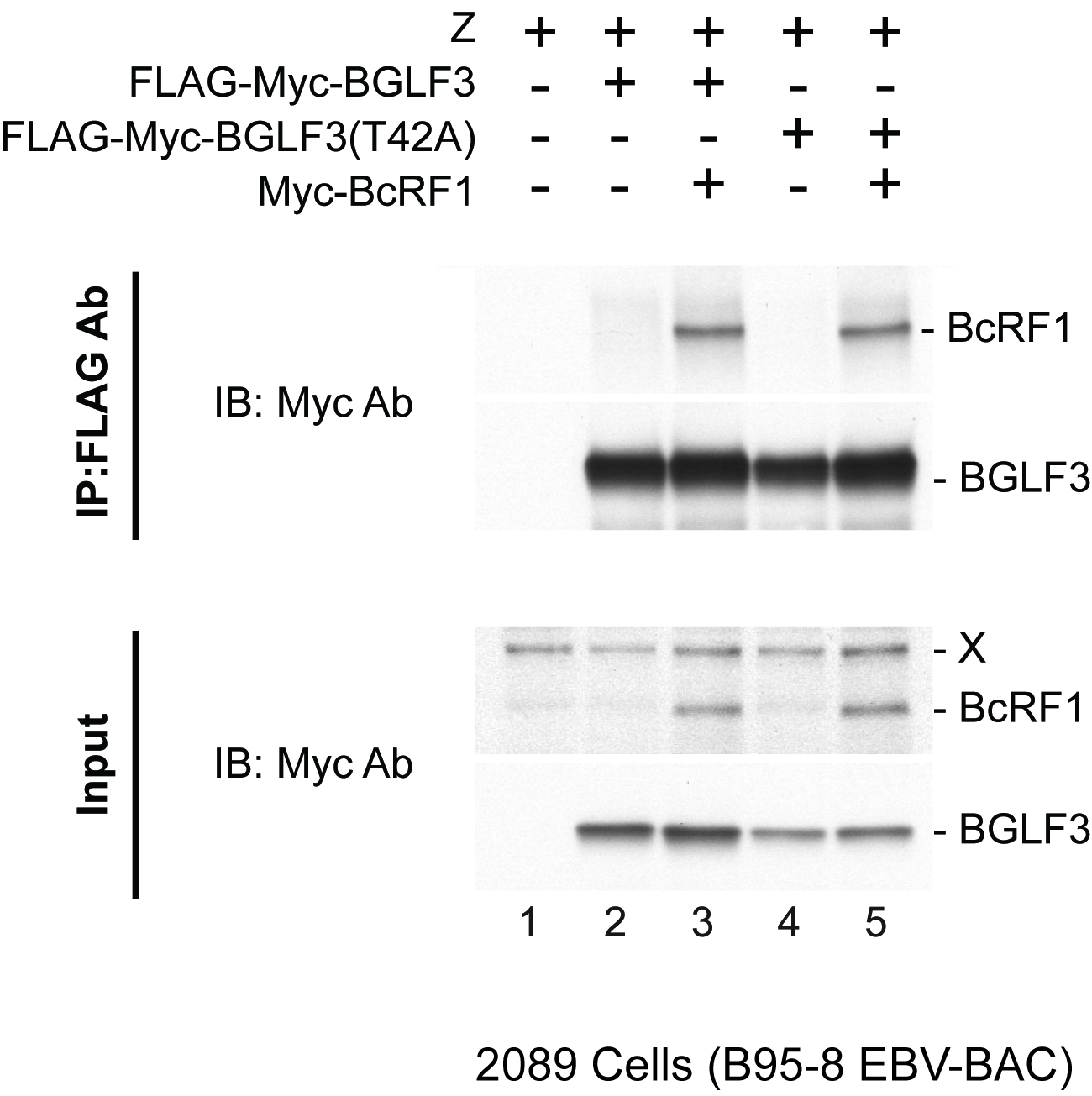

Supplement: S1 Fig — Comparing the capacity of wild type BGLF3 and BGLF3(T42A) to interact with BcRF1 during lytic infection. 2089 cells were transfected with FLAG-tagged BGLF3 or BGLF3(T42A) with and without the viral TATA box binding protein BcRF1. ZEBRA was co-transfected in all the cells to induce the lytic cycle. Co-immunoprecipitation was carried out using FLAG antibody crosslinked to agarose beads. BGLF3 and associated proteins were eluted using 3X FLAG peptide. Myc antibody was used to detect both BGLF3 and BcRF1. As shown in lanes 3 and 5, both wild type BGLF3 and BGLF3(T42A) interact with equal affinity to BcRF1. (TIF) [file ppat.1007980.s001.tif]
